# Effect of Selenium Supplementation on Biotin and Selenobiotin Concentrations in *Meyerozyma guilliermondii* and *Trichosporon cutaneum* Cells

**DOI:** 10.3390/molecules29235607

**Published:** 2024-11-27

**Authors:** Andrea Maria Patelski, Urszula Dziekońska-Kubczak, Agnieszka Nowak, Maciej Ditrych, Maria Balcerek, Katarzyna Pielech-Przybylska, Piotr Dziugan

**Affiliations:** 1 Institute of Fermentation Technology and Microbiology, Faculty of Biotechnology and Food Sciences, Lodz University of Technology, Wolczanska 171/173, 90-530 Lodz, Poland; urszula.dziekonska-kubczak@p.lodz.pl (U.D.-K.); agnieszka.nowak@p.lodz.pl (A.N.); maria.balcerek@p.lodz.pl (M.B.); katarzyna.pielech-przybylska@p.lodz.pl (K.P.-P.); 2 Department of Environmental Biotechnology, Faculty of Biotechnology and Food Sciences, Lodz University of Technology, Wolczanska 171/173, 90-530 Lodz, Poland; piotr.dziugan@p.lodz.pl

**Keywords:** biotin, selenobiotin, selenium, cutaneum, guilliermondii

## Abstract

Numerous studies have demonstrated the efficacy of selenium compounds in preventing and treating lifestyle-related diseases such as cancer and cardiovascular disorders. The formulation of selenium-enriched supplements for humans and animals, particularly those containing selenium yeast, is highly advantageous. These products are rich in organic selenium derivatives, showing significantly higher bioavailability than inorganic forms of selenium. A particularly promising selenium analogue of sulphur-containing compounds is selenobiotin. The literature indicates that *Phycomyces blakesleeanus* and *Escherichia coli* strains can synthesise this compound. This research aimed to evaluate the effect of selenium supplementation on the biosynthesis of biotin and selenobiotin in *Trichosporon cutaneum* and *Meyerozyma guilliermondii*. The results have the potential to advance biotechnological approaches for the production of selenobiotin for various applications. A method based on affinity chromatography was used to quantify selenobiotin. The results confirmed that both yeast strains could synthesise selenobiotin in addition to biotin. In *M. guilliermondii* cells, selenobiotin accounted for up to 17.3% of the total biotin vitamer fraction. In comparison, in *T. cutaneum* cells, it accounted for up to 28.4% of the sum of biotin and its analogues. The highest levels of selenobiotin were observed in cells cultured with selenomethionine.

## 1. Introduction

Yeast has accompanied humans for millennia. Although the use of these microbes was initially related to fermentation processes (winemaking, brewing, distilling and breadmaking) [1,2,3,4,5], the last 100 years have brought many other industrial applications of yeast strains, such as the production of bioethanol, biodiesel, enzymes, sweeteners and hormones [6,7,8,9,10,11,12]. Probiotic yeasts (*Saccharomyces boulardii*) have been produced for almost 70 years [13,14], and various yeast species have been cultured for feed purposes for practically 100 years [15,16,17,18,19,20,21]. The food applications of yeast are linked not only to the high content of easily digestible protein in the yeast biomass but also to the B vitamin content. The ability of yeast to convert non-organic compounds of chromium, magnesium, iron, zinc or selenium into organic derivatives, which are easily absorbed by animal and human organisms, is also highly desirable [22,23,24,25,26,27]. This allows yeast biomass to be used as a carrier for various micronutrients. As a result of numerous scientific studies confirming the salutary effects of selenium on our health, projects have emerged for the purposeful introduction of selenium compounds, especially its organic forms, into food. Studies have shown the effectiveness of selenium compounds in preventing and treating civilisation diseases, such as cancer and cardiovascular disease [22,23,28,29,30,31]. It is interesting and profitable to construct preparations for humans and animals containing selenium yeast, rich in selenium organic derivatives characterised by much higher bioavailability than inorganic selenium. Examples of patents from countries worldwide justify addressing this topic [32,33,34,35,36].

Because of the structural similarity of selenium and sulphur atoms, many sulphur compounds have their selenium derivatives. The most known selenium bio-compounds are selenomethionine and selenocysteine—amino acids which are derivatives of methionine and cysteine where the sulphur atom is replaced by selenium due to the structural similarity of these two molecules, and their combined occurrence in nature. Selenium derivatives are expected, by structure, to substitute sulphur-containing analogues in biological structures and actions. However, it should also be stated that these compounds, due to the selenium atom presence, are at least potential sources of selenium, with specific and different properties compared to their sulphur-containing analogues. Due to the possibility of intolerance to preparations containing live yeast cells, it may be advantageous to obtain preparations containing biologically active selenium metabolites, such as selenium amino acids or, for example, selenobiotin [37,38,39,40,41].

A very interesting selenium derivative of its sulphur analogue is selenobiotin. Very few papers have been published on this compound, its biosynthesis and its biological functions. Lindblow-Kull et al. mentioned that selenobiotin may be produced by the *Phycomyces blakeesleanus* strain [42]. Tse Sum Bui et al. [43] noted that the *Escherichia coli* strain may also biosynthesise it. Piffeteau et al. described that selenobiotin may substitute biotin in the catalysis of pyruvate carboxylase’s action [44]. Besides these papers, very little information about the biosynthesis and properties of this exciting molecule have been published. Some selenobiotin properties are predictable and connected with its structural similarity to biotin. However, do the specific biological functions of this compound result from the presence of selenium atoms in the molecule? Reliable strains and methods for producing this compound are needed to evaluate it.

Biotin is a heterocyclic compound consisting of ureido and tetrahydrothiophene rings. The valeric acid side chain is appended to the tetrahydrothiophene ring. The ureido ring contains the –N–CO–N– group. It serves as the specific carbon dioxide carrier in the carboxylation reactions of carboxylases and is involved in the decomposition of carbohydrates, synthesis of fatty acids and gluconeogenesis [45,46]. Apart from these primary roles, biotin also serves in less-known sites of metabolism, like chromatin stabilisation and gene expression [47]. Apart from biotin, some of its precursors and derivatives, like selenobiotin, may serve as the biotin for auxotrophic to biotin organisms, including microbial strains. It is known that for *Saccharomyces cerevisiae* DSM 2125—an auxotrophic strain used for biotin assays—desthiobiotin and some aminopelargonic acids may serve as growth activators [48,49,50,51,52]. Thus, when the biological assay is performed with the use of the yeast strain mentioned above, it is more correct to use the description “biotin vitamers” for the sum of biotin and its biologically active precursors and derivatives, like selenobiotin [42,44,52].

*Meyerozyma guilliermondii* and *Trichosporon cutaneum* are often used in laboratory and industrial animal feed research due to their broad nutritional capacity and ability to efficiently grow in feedstocks from waste materials such as hydrolysates of lignocellulosic materials. *Meyerozyma guilliermondii* attracted industrial interest at the beginning of the 20th century following the finding that certain strains could efficiently produce riboflavin. Subsequent research focused on their capability to absorb heavy metals. More recently, studies of *M. guilliermondii* have focused on its potential for xylitol production and degradation of polycyclic aromatic hydrocarbons [53]. This yeast has also demonstrated efficacy in inhibiting moulds responsible for fruit spoilage, leading to its inclusion in various studies on non-chemical post-harvest treatments and proposals for using its toxins for fruit protection [54]. *M. guilliermondii* antimicrobial properties have also been reported for the protection of bread [55], olives [56] and wheat [57]. These strains were also successfully tested for biomass cultivation using sugar beet pulp and potato waste peels [15,16]. The improved lactation and increase in immune parameters of sows and litters were also observed after feeding them with *M. guilliermondii* cells [58]. The positive growth performance of the rainbow trout feed with biomass of this yeast was also noted [59]. *Trichosporon cutaneum* is known for its ability to grow on pentose-containing media. Compared to *Yarrowia lipolytica*, *T. cutaneum* metabolises pentose sugars more efficiently and tolerates inhibitors [60], making it ideal for lipid production from lignocellulosic biomass. However, its dimorphic switch between yeast and hyphal forms during fermentation affects broth rheology and mass transfer, thereby affecting lipid yield [61,62]. These features are promising for further applications of these strains in various biotechnological processes, including obtaining yeast biomass enriched with selenium.

This study aimed to evaluate the influence of selenomethionine, selenocysteine, sodium selenate and sodium selenite on biotin and selenobiotin biosynthesis by *Trichosporon cutaneum* LOCK 0254 and *Meyerozyma guilliermondii* ATCC 6260. Biotin vitamer content, including selenobiotin, in the cell hydrolysates was assessed using the auxotrophic to biotin test strain *Saccharomyces cerevisiae* DSM 2155. The results may be crucial for future developments in the biotechnological production of selenobiotin for various purposes.

## 2. Results and Discussion

### 2.1. Effect of Selenium Supplementation on Biomass Yield, Selenium Accumulation and Biotin and Its Vitamers in Yeast Cells

The purpose of using a “0S” medium for the experiments was to increase the selenium/sulphur ratio in the medium, which, we assumed, should increase the selenium incorporation during the stage of sulphur or selenium addition to the desthiobiotin molecule and formation of the biotin molecule. Lack of biotin in the cultivation medium aimed toward forcing tested yeast strains (biotin prototrophs) to synthesise the biotin/selenobiotin molecule de novo, not just to incorporate the sulphur-containing biotin molecule from the medium to the enzymatic complexes of carboxylases. In the condition of a low concentration of S atoms and high presence of Se forms, we expected to increase the yield of selenobiotin formation. As a reference for the Se-substituted 0S media, the 0S medium was supplemented with potassium sulphate to supply the necessary amount of sulphur for biotin formation. 

The biomass concentration was also assayed after cultivation (Table 1).

The results presented in the table above mainly indicate that the biomass concentration was significantly (*p* < 0.05) dependent on the form of selenium used for supplementation. Preliminary unpublished experiments allowed us to establish the 50 μM/L dose as one that only slightly inhibited biomass growth; however, it is readily apparent that changing the form from sodium selenite to selenomethionine at the same molar dose resulted in a decrease in final biomass yield (DM) from 9.063 ± 0.055 g/L to 5.232 ± 0.216 g/L. The lowest biomass yield of 4.937 ± 0.293 g/L was observed in the medium with the addition of 50 μM/L selenourea. Concerning the reference medium, which was not supplemented with selenium, this decreased by about 48%. In comparison, for the application of sodium selenite at the same dose, the decrease was only about 5%. The total selenium concentration in the biomass obtained was a derivative of selenium supplementation and, like the concentration of dry cell mass, was significantly dependent on the form of selenium (*p* < 0.05). The minimum selenium concentration (12.239 ± 0.353 μg/g DM) was recorded after culture with sodium selenite supplementation. In contrast, the maximum, equal to 32.143 ± 1.453 μg/g DM, was determined in biomass supplemented during culture with organic selenium in the form of selenomethionine. The potential selenomethionine concentration in a selenium-free medium was below the detection threshold for the method used, which is consistent with the logic of the planned experiment. One of the objectives of the designed experiments was to determine the effect of selenium supplementation on the formation of biotin (and its derivatives), firstly to establish a possible correlation for purely cognitive reasons but also to determine the potential optimal form of sodium selenite, allowing the highest, under the given conditions, amount of biotin (and potentially selenobiotin) molecules in the biomass of cultured cells. With *M. guilliermondii* yeast and a dose of 50 μM Se/L medium, no clear correlation could be observed between the form of selenium (organic vs. inorganic) and the formation of biotin and its vitamers. However, it was observed that with selenium supplementation, the content of biotin and analogues ranged from 10.480 ± 0.324 μg/100 g DM to 12.091 ± 0.555 μg/100 g DM, while biomass grown in the reference medium without selenium supplementation contained only 8.413 ± 0.231 μg biotin per 100 g dry weight. 

Table 2 shows the results of biomass and biotin vitamer content in the *T. cutaneum* biomass cultivated in Se-enriched media.

The results of the preliminary cultivation studies with different concentrations of organic and non-organic forms of selenium allowed us to determine that the *Trichosporon cutaneum* strain was more resistant to the presence of selenium in the medium, which allowed us to consider the two selenium doses of 50 μM/L and 350 μM/L as representative for further studies. Although the reference biomass concentration after culture in the medium with 50 μM K_2_SO_4_ resulted in only 6.116 ± 0.132 g DM/L, which was about 30% lower than that obtained for the yeast *M. guilliermondii* (9.578 ± 0.433 g DM/L), the strain *T. cutaneum* was able to grow at a dose of 350 μM/L for different forms of selenium, reaching final biomass concentrations ranging from 1.828 ± 0.055 for the 350 μM/L selenomethionine-supplemented medium, to 5.234 ± 0.178 g DM/L for the 350 μM/L selenourea-supplemented medium. As with the *M. guilliermondii* strain, a significant (*p* < 0.05) decrease in biomass yield was observed when the medium was supplemented with 50 μM/L selenium and a substantial (*p* < 0.05) decrease in cultured cells was observed when supplemented with 350 μM/L selenomethionine. The yeast biomass of *T. cutaneum* cultured in the presence of 50 μM/L selenium ranged from 37.434 ± 3.394 μg Se/g DM (for sodium selenate) to 188.064 ± 6.935 μg Se/g DM (for selenomethionine), and these values were several times higher than those presented for *M. guilliermondii* cultured with the same selenium supplementation. The properties of the *Trichosporon cutaneum* strain also allowed it to grow in 350 μM Se/L, which resulted in a biomass very rich in selenium (284.134 ± 5.811 μg/g DM to 909.900 ± 22.261 μg/g DM). As with *M. guilliermondii* yeast, it was observed that the organic form of selenium resulted in a higher selenium content in the biomass of the cultured yeast. Although it is difficult to find results for selenium accumulation by comparable strains under similar conditions, our results can be compared with those of Zhang et al. [63], who, after cultivating *C. utilise* biomass with sodium selenite at 15 mg Se/L, obtained 13.35 g DM/L cells containing 1010 μg Se/g DM. Highly selenium-enriched yeast biomass of *Candida utilis* was also obtained by Kieliszek et al. [64], where after 48 h of culture in a medium supplemented with 30 mg Se/L (Na_2_SeO_3_), 14.1 g DM/L biomass containing approximately 1800 μg Se/g DM was isolated. Yang et al. [65] obtained 905.2 μg Se/g DM and 984.7 μg Se/g DM in *C. utilis* biomass in a medium supplemented with 15 mg/L Na_2_SeO_3_ in a batch and fed-batch system, respectively. Kieliszek et al. [66] conducted cultures of *S. cerevisiae* yeast, among others, and obtained almost 7 g of yeast DM/L of the medium; this biomass contained 800 μg Se/g DM. Martiniano et al. [67], by culturing *S. cerevisiae* yeast in a medium made of corn bran supplemented with molasses and 10 mg/L sodium selenite, obtained about 9 g DM/L containing 236.93 μg Se/g DM. Podgórska and Bujak [68] conducted an interesting study by culturing *S. cerevisiae* and *C. tropicalis* yeast in various selenium concentrations. They recorded a substantial decrease in the biomass concentration of *Saccharomyces cerevisiae* yeast, from 9.6 g DM/L in a control medium to 2.9 g DM/L in a medium supplemented with inorganic selenium at a dose of 2 μg Se/mL. They also observed a similar effect for the *Candida tropicalis* strain, reporting a decrease in biomass yield from 12.8 g DM/L (control medium without selenium) to 3 g DM/L in a medium with 2 μg Se/mL. The biomass of the selenium yeast they obtained contained 0.69 mg Se/g DM (*S. cerevisiae*) and 0.66 mg Se/g DM for *C. tropicalis* yeast [68]. An interesting study on obtaining selenium-enriched microbial biomass was conducted by Diowksz et al. [69]. Using selenium dioxide at doses of 1–20 μg Se/mL of medium, they obtained yeast *Saccharomyces cerevisiae* biomass containing from 2 to 364 μg Se/g DM. Marinescu et al. [70], using malt wort supplementation with sodium selenite in concentrations ranging from 30 to 180 μg/mL, obtained the yeast biomass of *Saccharomyces uvarum* containing 0.6–2.2 mg Se/g DM. Demirci and Pometto reported that using Na_2_SeO_4_ at a dose of 280 mg/L in continuous cultivation of *S. cerevisiae* resulted in a very low biomass yield close to 0.7 g/L, and the selenium content of the biomass was 687 μg/g DM. Compared to the experiment where sodium selenite at the dose of 690 mg/L was used, this content of selenium in the biomass was found to be 1904 μg/g DM and the production efficiency of yeast cell biomass was 1.8 g/L [38]. In the present study, the analysis of the content of biotin and its active derivatives in *T. cutaneum* biomass showed that the concentration of these compounds ranged between 133.607 ± 5.112 μg/100 g DM (after culture supplemented with 50 μM/L selenomethionine) and 183.686 ± 7.454 μg/100 g DM (after culture supplemented with 350 μM/L selenomethionine). Of note is the overall, more than 10-fold, higher concentration of biotin vitamers in the biomass of *Trichosporon cutaneum* yeast compared to the biomass of *M. guilliermondii* cells. The ranges of biotin content in yeast cells obtained by us (10.48–183.686 μg/100 g DM) are consistent with those presented in recognised books on yeast technology edited by Reed and Nagodawithana [71]. Lipińska et al. [72] report that the biotin content of *S. cerevisiae* cells is 34–36 μg/100 g DM. Oura et al., in their numerous papers on yeast and baker’s yeast production, cite for *S. cerevisiae* yeast a range of 30–200 μg/100 g DM while specifying that during industrial yeast propagation, the intracellular biotin content decreases in successive culture stages, which are related to the level of supplementation of this vitamin to the culture medium [50,51,73]. Suomalainen et al. [74] cite a wide range of 40–110 μg of this vitamin in 100 g DM of baker’s yeast. Also, the website https://www.feedtables.com (accessed on 9 October 2024) provides data for the composition of animal feeds and gives a biotin content of 120 μg/100 g DM in yeast dry matter, but does not specify which yeast is meant [75]. Also interesting is the clear trend (*p* < 0.05) of a positive correlation between an increase in the concentration of biotin and its derivatives in the cultured biomass and an increase in selenium supplementation, which should be verified and clarified by more detailed biochemical studies in this field; however, this is beyond the scope of our planned research.

**Figure 1:**
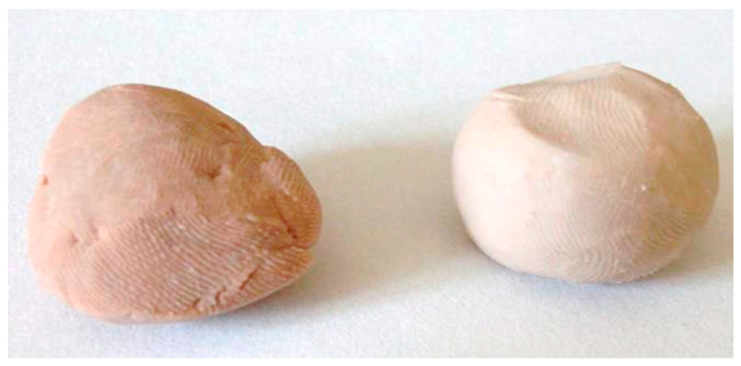
Pink-reddish colour of the *Trichosporon cutaneum* ATCC 6260 biomass obtained with 350 μM/L of sodium selenite (Se^4+^)—on the left. On the right is the biomass obtained after cultivation in a reference medium (without selenium supplementation) (author: A.M. Patelski).

Figure 1 compares two portions of pressed wet biomass of the yeast *Trichosporon cutaneum*, grown with and without selenium.

Due to the toxic effects of high selenium concentrations on yeast cell metabolism [76,77], obtaining selenium-rich biomass requires finding the ‘golden mean’ between the conditions for maximum selenium accumulation in the cell and the desired high biomass yield. Yeast cells defend themselves against the toxic effects of selenium in several different ways. Literature data report increased production of glycogen and trehalose in selenium-containing media [78]; this can be observed as the appearance of granulation in the cytoplasm, as reported by Podgórska and Bujak [68], also supported by Gharieb et al. [79]. Another interesting visual phenomenon is the reduction of oxidised, active forms of selenium to low-toxic elemental Se^0^, giving the biomass a pink or red colour. This phenomenon was also observed in our study. Similar observations were made by Martiano et al. [67], Kieliszek et al. [78] and Diowksz and co-workers [69]. The biomass of selenium yeast obtained by us was also marked by a specific garlic–onion smell, which, according to the literature reports, may be the result of another method of cell detoxification from selenium, involving the transfer of its atoms to the volatile dimethyl selenide released not only from the cell but also from the close vicinity of the cell. This strategy was reported by Mapelli et al. [80], Kieliszek et al. [81] and Ohta et al. [82]. Also, dimethyl selenide was reportedly present in human breath and sweat exposed to toxic selenium concentrations [83]. In light of our study, this pathway may be supportive in the biosynthesis of selenobiotin, as some studies aiming to identify the sources of sulphur in biotin (and selenium in selenobiotin) report a strong effect of sodium sulphide (or sodium selenide) in stimulating the biotin synthase-catalysed insertion reaction of sulphur (or selenium) into desthiobiotin to form the cyclic biotin (or selenobiotin) ring [43,84].

The results obtained during this first part of the research helped us to select the most promising dose of selenium, aiming toward the most efficient biosynthesis of biotin and selenobiotin at reasonably high biomass concentrations.

### 2.2. Selenobiotin Content in the Biomass of the Tested Yeast Strains Cultivated with Various Selenium Supplementation

The following tables show the selenobiotin contents of the yeast biomass of *Meyerozyma guilliermondii* (Table 3) and *Trichosporon cutaneum* (Table 4) grown in media supplemented with different forms of selenium.

Avidin immobilised on agarose beads was used to isolate selenobiotin and biotin from the total pool of biotin vitamers. The properties of the beads allowed for the specific isolation of only biotin and selenobiotin from the purified and concentrated cell hydrolysates analysed. In isolates eluted from a highly specific avidin-coated resin containing only biotin and its selenous derivative, the selenium content was determined, corresponding in molar terms to the amount of selenobiotin molecules. Control samples were obtained from the biomass without selenium supplementation. Also, pure biotin in aqueous solution was used as a control sample. The sum of selenobiotin and biotin was determined microbiologically using the auxotrophic test strain *S. cerevisiae* DSM 2155. 

Based on the selenium content of the concentrated isolates, the selenobiotin content was calculated and expressed as its concentration in the biomass. These results were also expressed as a percentage of the biotin and selenobiotin sum isolated using avidin–agarose columns. The selenobiotin content of *M. guilliermondii* yeast cells ranged from 1.564 ± 0.051 μg/100 g DM (for cultures with 50 μM/L sodium selenate present) to 2.000 ± 0.046 μg/100 g DM (for biomass obtained in medium supplemented with 50 μM/L selenomethionine). In the biomass of *Meyerozyma guilliermondii* cultured with sodium selenate, selenobiotin accounted for 10.2%, and in the biomass with selenomethionine, it accounted for as much as 17.3% of the total sum of biotin and selenobiotin present in the sample.

In contrast, the selenobiotin content in *T. cutaneum* yeast cells ranged from 30.165 ± 0.974 μg/100 g DM (for cultures with 350 μM/L sodium selenite) to 52.543 ± 2.616 μg/100 g DM, for biomass obtained in the medium supplemented with 350 μM/L selenomethionine. In the biomass of the *Trichosporon cutaneum* strain grown with sodium selenate, selenobiotin accounted for 21.3% of the total biotin and selenobiotin pool, whereas in cells of the same yeast grown with 350 μM/L selenomethionine supplementation, selenobiotin accounted for as much as 28.4% of the total biotin and selenobiotin. The content of the selenium derivative of biotin significantly (*p* < 0.01) depended on the yeast strain used. The selenobiotin concentration in the *Trichosporon cutaneum* yeast was 25–30 times higher than in the *Meyerozyma guilliermondii* biomass. Selenobiotin accumulation within a strain also depended on the form of selenium. When inorganic selenium was added to the culture medium, selenobiotin concentrations were significantly (*p* < 0.05) lower than for biomass obtained in the presence of organic selenium (selenourea, selenomethionine and selenocysteine). The highest selenobiotin concentrations (and corresponding contributions to the biotin and selenobiotin pool) were observed when the medium was supplemented with selenomethionine. This observation supports suggestions by Ma et al. [85] and Tse Sum Bui et al.’s early investigations [86] that the selenium in the selenobiotin ring is likely to be derived from selenomethionine molecules. So far, the formation of selenobiotin has been confirmed by Lindblow-Kull et al. [42] and Tse Sum Bui et al. [43] for *Phycomyces blakesleeanus* and *Escherichia coli*, but it must be recognised that the subject matter touching on selenobiotin biosynthesis is still niche. Therefore, our results can be considered a baseline for further work. 

Our results indicate the formation of a selenium derivative of biotin by the yeasts *Meyerozyma guilliermondii* and *Trichosporon cutaneum*. This suggests that selenobiotin biosynthesis is probably a common phenomenon based on the metabolic scales of biotin formation. In designing the culture supplementation with a broad spectrum of selenium forms, we wanted to check which of these would noticeably intensify selenobiotin formation under the conditions of a planned deficiency of sulphur (although it should be noted that minimal amounts of this element were introduced even with the inoculum cells, as were undefined, undiminished amounts of biotin). The results showing the contribution of selenobiotin to the total pool of biotin and its selenium derivative indicate that supplementation with organic forms of selenium significantly increased (*p* < 0.05) the formation of selenobiotin. This was most noticeably observed when selenomethionine was added to the medium. It can also be seen that the proportion of selenobiotin in the pool of biotin vitamers in the biomass of the *Trichosporon cutaneum* was twice as high as that determined in the cells of *Meyerozyma guilliermondii*, and this is probably due to the effect of the resistance of the first strain to selenium, which allowed it to grow at several-fold higher selenium concentrations. The origin of the sulphur atoms incorporated by biotin synthase into the selenobiotin molecule has been the subject of scientific interest for about 30 years. So far, using radioisotope studies, among others, it has been established that biotin synthase requires S-adenosylmethionine for its activity and that the sulphur atom itself occurs in the enzyme as a linkage to the [2Fe-2S]^2+^ space cluster [43,85,86,87,88]. One recent report by Lachowicz et al. [89] indicates the existence of a biotin synthase with axillary clusters in the form of 4Fe-5S structures for incorporating sulphur into desthiobiotin. The authors suggest that sulphur atoms can enter the environment (and later the cell) through selenomethionine or selenocysteine. Several extensive studies have been carried out by Tse Sum Bui et al. [43,84,86], in which a strong effect of Na_2_S supplementation on biotin formation was demonstrated, as well as studies on its selenium counterpart Na_2_Se showing the appearance of iron–selenium clusters [2Fe-2Se]^2+^, in parallel to ‘traditional’ iron–sulphur clusters [43]. 

In light of the literature data and the findings of our study, it seems highly probable that any selenium-containing compound that can be transformed into the selenide form, for example, as a result of the detoxification mechanism mentioned earlier, can donate selenium atoms to form selenobiotin molecules, which should be investigated in more detailed and extensive studies in this area. 

## 3. Materials and Methods

### 3.1. Microbial Pure Cultures

The strains used in the experiments were *Trichosporon cutaneum* LOCK 0254 and *Meyerozyma guilliermondii* ATCC 6260—both known as able to synthesise biotin. The *Saccharomyces cerevisiae* DSM 2155 strain was used for the biotin vitamers assay—assessing the sum of the biotin, selenobiotin and its biologically active precursors, like desthiobiotin and aminopelargonic acids.

### 3.2. Paper Discs for Microbiological Biotin Assay

Schleicher & Schuell^®^ (Global Life Sciences Solutions, Warsaw, Poland) antibiotic-assay paper discs, with a 12 mm diameter, were used for the biotin microbiological assay.

### 3.3. Selenium Sources

The inorganic sodium selenate, Na_2_SeO_4_, and sodium selenite, Na_2_SeO_3_, and the organic selenomethionine, selenocysteine and selenourea, all produced by Sigma Aldrich (Warsaw, Poland), were used as selenium sources.

### 3.4. Avidin–Agarose Resin

PierceTM (#20228) Monomeric Avidin Agarose (Fisher Thermo Scientific, Warsaw, Poland) was used to separate biotin and selenobiotin from the initially purified cell hydrolysates.

### 3.5. Medium for Inoculum Preparation

The commonly known YPD medium was used for inoculum preparation. Yeast cells were transferred with the inoculating loop into 1 L round-bottomed flasks containing 150 mL of YPD medium, pH 5.2 ± 0.1, sterilised in the autoclave at 121 °C for 21 min. Shaken cultivations were performed for 48 h at 32 ± 1 °C (Reciprocal Shaker Eberbach E5900, Belleville, NJ, USA). Subsequently, the yeast cells were centrifuged (Laboratory Centrifuge MPW380R, Warsaw, Poland) at RCF 5000 g/10 min, thrice washed with sterile deionised water, and suspended in 0.9% NaCl solution. Such suspensions were used as the inoculum source for specific cultivations in selenium-containing media. The cell concentration was assayed spectrophotometrically (Spectrophotometer Rayleigh Analytical Instrument, Beijing, China) at 540 nm, and the results were expressed in g of dry matter (DM)/L calculated from the prepared standard curve. 

### 3.6. Medium for Microbial Biotin Assays

Our experiments successfully used the “Oura biotin-free” medium [73] to assay biotin and selenobiotin with the test strain *Saccharomyces cerevisiae* DSM 2125. This medium was composed by mixing the S1–S4 solutions: 

S1: glucose 50 g; KCl 0.12 g; NaCl 0.06 g; CaCl_2_∙2H_2_O 0.09 g; MgCl_2_∙6H_2_O 0.52 g; agar 20 g; and distilled water 600 mL;

S2: (NH_4_)_2_SO_4_ 12 g; Ca-pantothenate 6.25 mg; myoinositol 125 mg; thiamine 5 mg; pyridoxine 6.25 mg; nicotinic acid 5 mg; S4 stock solution 1 mL; (NH_4_)_2_Fe(SO_4_)_2_∙6H_2_O 0.035 g; and distilled water 400 mL;

S3: H_3_PO_4_ 85% 1.6 mL;

S4: MnSO_4_∙H_2_O 3.8 g; CuSO_4_∙5H_2_O 0.5 g; ZnSO_4_∙7H_2_O 2.3 mg; CoSO_4_∙7H_2_O 2.3 mg; Na_2_MoO_4_∙2H_2_O 3.3 mg; H_3_BO_3_ 7.3 mg; KJ 1.7 mg; NiSO_3_∙6H_2_O 2.5 mg; and distilled water 1000 mL.

The S1 and S4 solutions were sterilised in an autoclave (121 °C/21 min). S3 was not sterilised, while S2 was sterilised by filtration using a 0.22 μm pore sterile filter.

For final medium preparation, the S1–S3 constituents were combined in the sterile flasks.

### 3.7. Medium for Examining Selenobiotin Biosynthesis

To evaluate the impact of the selenium form and dose on the biosynthesis of selenobiotin, our research group proposed a mineral medium as a potential solution. This medium, which we have designated the “0S” biotin-free medium, is a modified version of the Oura medium (as previously mentioned) in which sulphur salts have been removed. To obtain this medium, the MS1 and MS2 solutions were mixed, the pH was corrected to 5.6 ± 0.1, and the whole medium was sterilised by filtration (0.22 μm pore filter).

MS1: ferric ammonium citrate—[(NH_4_)_5_[Fe(C_6_H_4_O_7_)_2_] 0.1 g; NH_4_NO_3_ 29.03 g; KH_2_PO_4_ 6.45 g; MA* solution 10 mL, MC** solution 1 mL; and distilled water 1600 mL;

MS2: glucose 100 g; Ca-pantothenate 12.5 mg; myoinositol 250 mg; thiamine 10 mg; pyridoxine 12.5 mg; nicotinic acid 10 mg; agar 40 g; and distilled water 400 mL;

MA* solution: KCl 47.7 g; NaCl 25.5 g; CaC_l2_∙2H_2_O 36.7 g; MgCl_2_∙6H_2_O 20.9 g, and distilled water 2000 mL;

MC** solution: MnCl_2_∙8H_2_O 17.8 g; CuCl_2_∙2H_2_O 1.4 g; Zn(CH_3_COO)_2_∙2H_2_O 6.9 g; Co(NO_3_)∙6H_2_O 0.00985 g; Na_2_MoO_4_∙2H_2_O 0.013 g; H_3_BO_3_ 0.029 g; KJ 0.007 g; NiCl_2_∙6H_2_O 0.0095 g, and distilled water 2000 mL.

The MS1 and MS2 fractions were sterilised in an autoclave (121 °C/21 min), and the MA and MC fractions were sterilised by filtration using a 0.22 μm pore sterile filter. For final medium preparation, the MS1 and MS2 constituents were combined.

For selenium supplementation, various selenium-containing compounds were added to this medium, and the range of the doses was 0–0.35 μM/L for the *T. cutaneum* strain and 0–50 μM/L for *M. guilliermondii.* These ranges were established for each specific strain by the unpublished preliminary results of the cultivations of these strains at a broad range of selenium doses (0–0.6 μM/L).

### 3.8. Cultivations

Cultivations of *Trichosporon cutaneum* LOCK 0254 and *Meyerozyma guilliermondii* ATCC 6260 were carried out as shaken ones for 48 h at 30 ± 1 °C, using a reciprocal shaker with 1 L flat-bottomed glass flasks containing 150 mL of the medium, closed with gauze plugs. 

### 3.9. Yeast Biomass Separation

After cultivation, the biomass was separated by centrifugation (RCF 5000 g/10 min). Subsequently, it was triple-rinsed with distilled water. After final centrifugation, it was collected in plastic containers and stored at −20 °C for further assays.

### 3.10. Biomass Concentration Assay

Yeast biomass was quantified by determining the cell dry mass using the thermogravimetric method. Cells were separated by centrifugation (Laboratory Centrifuge MPW380R, Warsaw, Poland) at RCF 5000 g/10 min, rinsed with distilled water and dried at 105 ± 1 °C (Suslab Bio 005 Laboratory Drier, Lodz, Poland) until a stable weight was reached. The results were expressed in g/L. 

### 3.11. Isolation of Crude Extracts of Biotin and Its Vitamers from Yeast Biomass

The procedure was proposed by Lezius et al. [90] and was performed as follows. A total of 10 g of yeast biomass was mixed with 40 mL of citrate buffer, 0.3 mL of 1% (*w/w*) freshly prepared water solution of reduced glutathione, 0.3 mL of 1% (*w/w*) of EDTA solution and 5 mL of 2% (*w/w*) papain in water solution. The mixture was left for 24 h at 37 °C. After this time, necessary for efficient enzymatic hydrolysis, the mixture was adjusted with water to the total volume of 100 mL. Then, it was filtered through a 0.45 μm filter. A total of 2 g of charcoal powder was added to the prepared liquid and mixed for 1 h. Then, the charcoal fraction with the absorber biotin and its derivatives was filtered using the Whatman 1 filter paper. Biotin and its derivatives were then rinsed out from the charcoal with the use of 3 portions of 50 mL mixture containing 25 mL of 90% (*v/v*) ethanol, 20 mL of water and 5 mL of 25% (*w/v*) of NH_4_OH. Biotin and its vitamer-containing solution were then concentrated to 6–7 mL using a vacuum rotary drier (p = 2 × 10^3^ Pa; temp = 60 °C). Then, the pH was corrected to 6–7 units, using a 25% solution of HCl; after this, the volume was filled with water to 10 mL. This prepared solution was stored for further analysis at −20 °C.

### 3.12. Biotin and Its Vitamers Assay in Yeast Cell Hydrolysates

Despite the implementation of methods based on the combination of HPLC and mass spectrometry [91,92,93] for the determination and speciation of biotin and biotin derivatives, our laboratory has been using methods based on the use of auxotrophic test microorganisms relative to biotin, such as the yeast *Saccharomyces cerevisiae* DSM 2155 [94] or the bacterium *Lactiplantibacillus plantarum* ATCC 8014 [95]. These procedures are still regarded as reference methods for qualitative and quantitative biotin assays in many fields [94,95,96,97,98]. 

**Figure 2:**
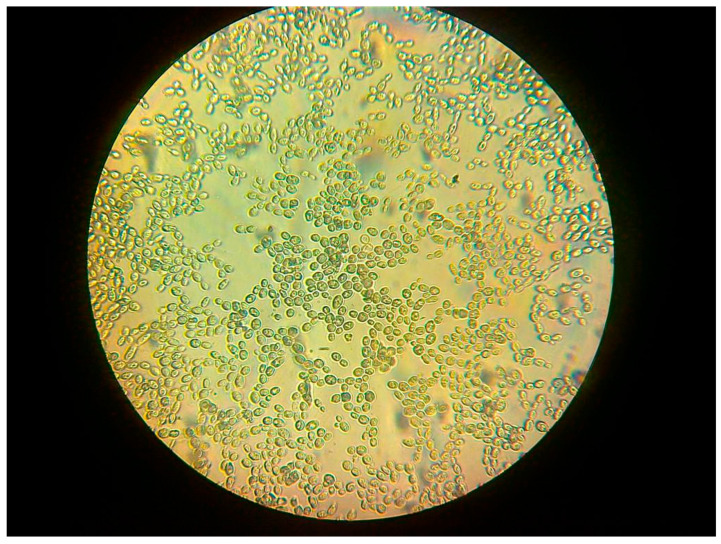
Microscopic view (×480) of *Saccharomyces cerevisiae* DSM 2155 cells (author: A.M. Patelski).

In our study, the total amounts of biotin and its biologically active derivatives were assayed using the biological method proposed by Genghof [94], with the Oura medium [73] and test strain *Saccharomyces cerevisiae* DSM 2155. Glass Petri dishes (diameter 15–17 cm) were sterilised at 140 °C for 5 h before this assay. Also, the paper discs (Schleicher & Schuell^®^ antibiotic-assay discs, 12 mm diameter) were sterilised under the same conditions. Before the assay, the Oura “biotin-free” medium with 2% agar (Difco) was prepared and cooled to approx. 40 °C and 5 mL of yeast cream (containing about 0.25 g DM of *S. cerevisiae* DSM 2155 cells) was mixed with the medium and poured on the bottom of the Petri dish. On the solidified medium surface, 5–8 antibiotic-assay paper discs were placed (soaked with standard solutions of biotin or with purified cell hydrolysates). Standard solutions were in the range from 0.0012 to 1.2 µg of biotin/mL. The upper dish was placed on the bottom, and plates were settled in the incubator (32 °C for 48 h). After this time, the growth zone diameters for standard biotin solutions were measured, and the biotin content in cell hydrolysates was calculated from the diameters of the growth zones around specific paper discs. The results were expressed as the biotin µg/mL of concentrate or as µg/g DM of yeast cells. Figure 2 shows yeast cells of the *Saccharomyces cerevisiae* DSM 2155 strain. 

**Figure 3:**
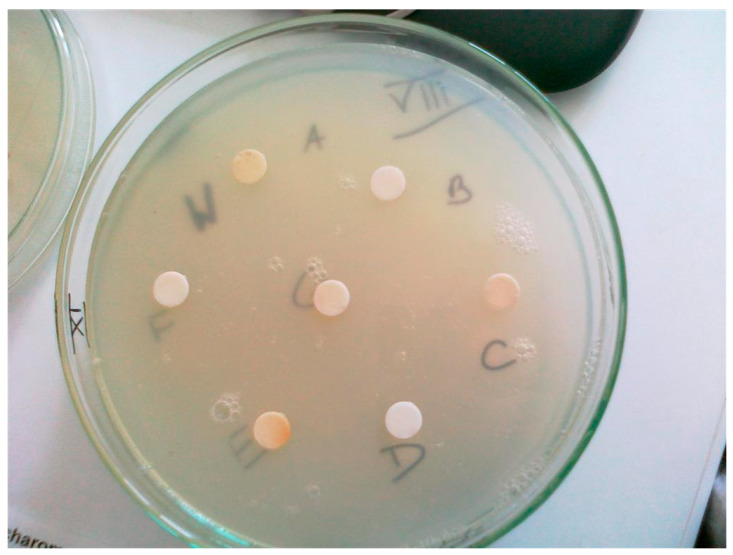
Petri dish before incubation with paper discs containing biotin (author: A.M. Patelski).

**Figure 4:**
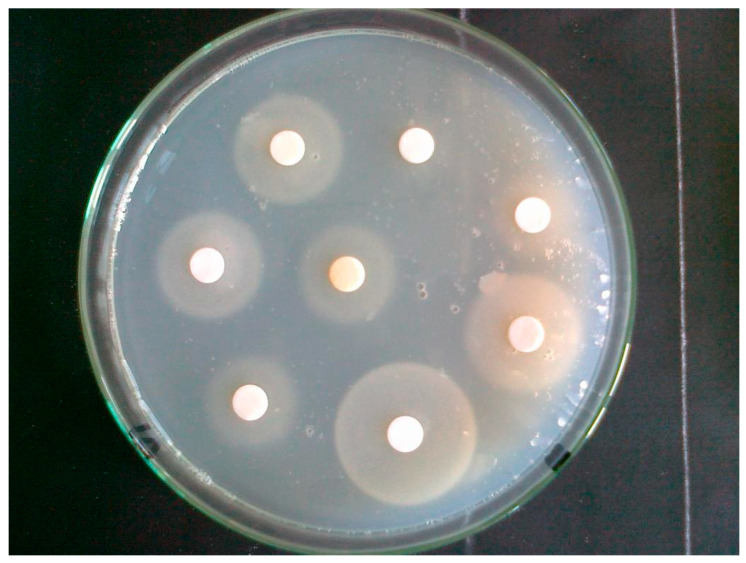
Petri dish after 48 h incubation. The growth zones of the auxotrophic to biotin test strain are visible around the paper discs. The zone diameter depends on the biotin concentration in the analysed solution (author: A.M. Patelski).

Figure 3 and Figure 4 show the appearance of the exemplary plates before and after culturing the test yeast *S. cerevisiae* DSM 2155 during biotin determination in hydrolysates of yeast cells cultured with various selenium compounds. The photos show the principle of the microbiological determination of biotin. On the surface of a biotin-free agar medium containing cells of the auxotrophic strain DSM 2155, paper discs immersed in a test or standard solution containing biotin (and/or its active biological derivatives) were placed. The growth zone after 48–72 h of culture was proportional to the biotin content of the disc. This method allows biotin and its derivatives to be determined using a simple microbiological method.

### 3.13. Selenobiotin and Biotin Isolation from Cell Hydrolysates

For selenobiotin and biotin isolation, the affinity chromatography method was used based on the selective and reversible binding of biotin and selenobiotin in the column packed with immobilised avidin. To prepare concentrated biotin and selenobiotin solutions from initially purified cell hydrolysates, the Pierce^TM^ Monomeric Avidin Agarose resin was used as a glass column packaging. Monomeric avidin agarose was initially manufactured to separate and purify biotinylated proteins, peptides and other macromolecules containing biotin molecules. In this resin, the monomeric avidin is immobilised on the surface of agarose beads. Using monomeric avidin instead of native tetrameric avidin enables biotin-containing substances’ specific but reversible immobilisation. It’s possible to rinse the immobilised biotinylated fractions at relatively mild conditions—glycine solution is used to rinse out the biotin-containing molecules from the resin (unlike the requirements when tetrameric avidin is used). The manufacturer claims that such resin may be recycled 10 times without significant affinity loss [99]. 

For purification of the biotin/selenobiotin fraction, a chromatographic column (8 mm internal diameter) was prepared and packed with 2 mL of avidin–agarose beads. The column was then rinsed with 30 mL of PBS buffer (mixture of 0.1 M sodium phosphate and 0.15 M sodium chloride in water, pH = 7). Subsequently, the column was rinsed with 5 mL of PBS buffer containing 2 mM of biotin to irreversibly saturate the beads’ places where avidin formed unwanted complex structures, distinguished by irreversible (one-time) binding of biotin molecules. In comparison, most of the avidin-coated surface is ready to bind biotin or biotinylated substances, and then these substances may be rinsed off the resin with a glycine solution. Then, a regeneration buffer (0.1 M of glycine, pH 2.8, volume 30 mL) was applied to the column to rinse biotin from places that reversibly bound the biotin molecules. Subsequently, the column was rinsed to remove residual glycine with PBS buffer (30 mL). The prepared column was ready for biotin/selenobiotin immobilisation from our previously prepared cell hydrolysate fractions. The solution for the separation of biotin and selenobiotin (3–5 mL) was passed through such a prepared column, and the effluent was thrice recycled to maximise the biotin/selenobiotin capture on avidin. After passing the sample volume through the column, it was rinsed with 50 mL of PBS buffer. Then, the immobilised biotin and selenobiotin from cell hydrolysates were rinsed with 30 mL of 0.1 M of glycine solution. After rinsing, the aqueous effluent passing out the column contained biotin/selenobiotin (and glycine—neutral for further biotin and selenium assays) from cell hydrolysates. Then, the successive hydrolysate samples were purified, or the column was preserved for use in the following days by filling it up with 0.01% sodium azide in PBS buffer. Before use, the sodium azide was rinsed out with 30 mL of PBS buffer. The vacuum facilitated liquid flow through the column (0.015–0.025 MPa). Fractions purified by avidin–agarose resin affinity were then vacuum evaporated to achieve the final volume of the sample, between 5–10 mL. Such prepared fractions were preserved by freezing (−20 °C) and stored for the biotin and selenium assay.

To confirm the applicability of the method, in addition to the samples obtained after hydrolysis of the yeast cells, test samples containing a mixture of (1) biotin and selenobiotin, (2) biotin, selenourea, selenomethionine and selenocysteine or (3) sodium selenate and sodium selenite were carried out.

**Figure 5:**
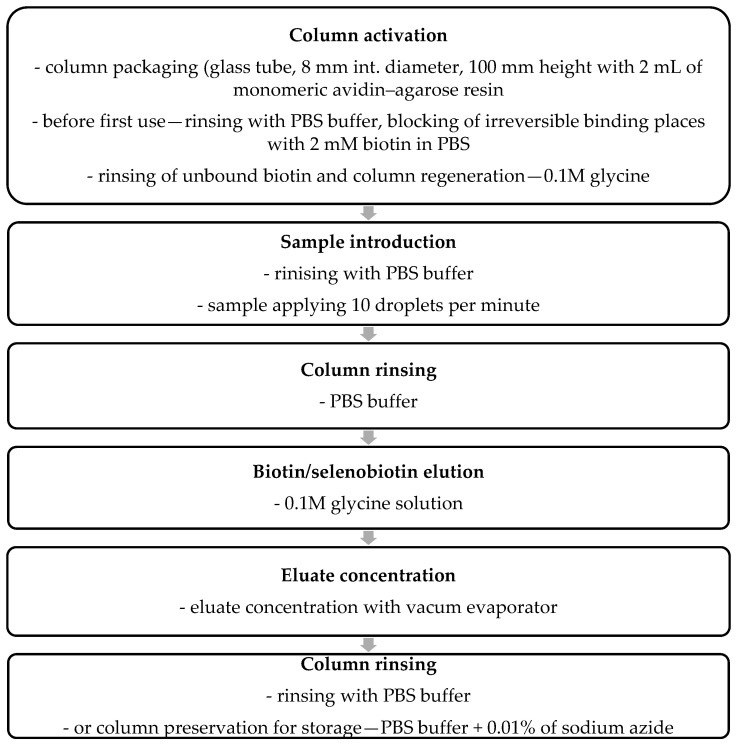
Scheme of biotin and selenobiotin isolation with the use of avidin–agarose resin.

The biotin/selenobiotin isolation scheme from initially purified cell hydrolysates is shown below (Figure 5).

### 3.14. Selenium Assay

To extract selenium before the primary assay, the samples were hydrolysed in the mixture of HNO_3_ and HClO_4_ (4:1 *v/v*) with a temperature cascade: 60 °C/12 h → 110 °C/1 h → 140 °C/0.5 h → 160 °C/0.5 h → 180 °C/1 h. The mixture was cooled down, and to reduce Se^6+^ to Se^4+^, HCl was added, and the mixture was kept at 100 °C for 30 min. After cooling the mixture to 20 °C, NH_2_-EDTA was added, and the pH was corrected to pH = 2 with NaOH; then, the hydrolysates were filled with 0.01 M HCl to a known volume, allowing calculation of the selenium concentration in the initial sample. 

For the selenium assay, we adopted the well-proven method proposed by Walkinson [100] and modified by Alftham [101,102]. Selenium was assayed spectrophotometrically using 2,3-diamino naphthalene (DAN) as a reagent. The complex of selenium and DAN was extracted using cyclohexane. Fluorescence was then measured using a spectrofluorometer at 377 nm (excitation) and 516 nm (emission). The results were calculated from the formula of the calibration curve prepared using the standard selenium solutions. The results of the analysed biomass were expressed as [μg Se/DM g].

### 3.15. Selenobiotin Assay in Purified Fractions of Cell Hydrolysates 

The sum of the biotin and selenobiotin in the hydrolysates purified with avidin–biotin affinity chromatography was assayed microbiologically with the standard method, based on the growth of the auxotrophic to biotin test strain *Saccharomyces cerevisiae* DSM 2155. In the same sample, selenium content was also assayed. With the assumption that 1 mole of selenobiotin contains 1 mole of selenium molecules, the selenobiotin presence in the total amount of biotin vitamers in the sample was calculated. 

### 3.16. Statistical Treatment

All assays were carried out in at least triplicate. Statistical analysis (variance analysis, SD determination, Student’s *t*-test at a significance level of a = 0.05) was done using the Origin 7.5 computer program. 

## 4. Conclusions

The results of our study confirm the possibility of selenobiotin biosynthesis by the yeasts *M. guilliermondii* and *T. cutaneum*. The latter strain grew in selenium concentrations six times higher than the yeast *M. guilliermondii*. The yeasts tested also differed significantly (*p* < 0.01) in the amounts of biotin and its derivatives formed. The yeast *T. cutaneum* contained 10–15 times more biotin and its analogues than the *M. guilliermondii*. Observations of biomass growth in a medium supplemented with inorganic and organic forms of selenium suggest that both strains exhibited the ability to undergo active metabolic detoxification of cells, involving reducing selenium to the methyl selenide and elemental selenium. The biomass we obtained was characterised by a reddish-pink colouration that can be elucidated due to the presence of atomic selenium and a garlic-onion odour derived from volatile methyl selenides. Using agarose beads with immobilised avidin allowed the purification of cellular hydrolysates and the isolation of biotin and selenobiotin. Our results, in combination with an analysis of the available literature, seem to indicate that selenobiotin biosynthesis is a common phenomenon as long as selenium is present in the environment and the cell can reduce it to Se^2+^, which allows selenium to be incorporated into the iron–selenium clusters necessary for active biotin biosynthesis by biotin synthase. In *Meyerozyma guilliermondii* cells, selenobiotin represented up to 17.3% of the total biotin vitamer fraction. At the same time, selenobiotin in *Trichosporon cutaneum* comprised up to 28.4% of the combined total of biotin and its analogues. The highest concentrations of selenobiotin were detected in cells cultivated with selenomethionine. Our results complement the scanty literature data on the formation of selenium derivatives of biotin. Based on a critical analysis of the results of this study, we plan to carry out a more extensive study of selenobiotin biosynthesis by microorganisms in the future. The preparative liquid chromatography and mass spectrometry will be used to analyse the selenium accumulation and its forms, together with a crystallographic study of selenobiotin formed by the microbes. These results may also serve as a starting point for developing research aimed at efficient methods of selenobiotin biosynthesis by strains known as extracellular biotin producers.

## Figures and Tables

**Table 1:** Biomass and biotin vitamer concentrations in *Meyerozyma guilliermondii* ATCC 6260 cells cultivated with selenium supplementation in a 0S medium.

Selenium Form	Se Dose [μM/L]	Biomass Concentration [g DM/L]	Se in Biomass [μg/g DM]	Biotin Vitamers [μg/100 g DM]
Selenite (Se^4+^)	50	9.063 ± 0.055	12.239 ± 0.353	10.48 ± 0.324
Selenate (Se^6+^)	50	8.473 ± 0.305	13.205 ± 0.298	12.933 ± 0.470
Selenourea	50	4.937 ± 0.293	28.989 ± 0.831	12.091 ± 0.555
Selenocysteine	50	6.187 ± 0.308	27.318 ± 1.220	10.615 ± 0.530
Selenomethionine	50	5.232 ± 0.216	32.143 ± 1.453	11.192 ± 0.192
Reference with 50 μM of K_2_SO_4_ (S^6+^)	0	9.578 ± 0.433	n.d. ^1^	8.413 ± 0.231

^1^ n.d.—not detectable

**Table 2:** Biomass and biotin vitamer concentrations in *Trichosporon cutaneum LOCK 0254* cultivated with selenium supplementation in a 0S medium.

Selenium Form	Se Dose [μM/L]	Biomass Concentration [g DM/L]	Se in Biomass [μg/g DM]	Biotin Vitamers [μg/100 g DM]
Selenite (Se^4+^)	50	5.995 ± 0.183	67.261 ± 2.087	135.590 ± 6.281
	350	3.416 ± 0.064	830.782 ± 19.139	154.680 ± 5.070
Selenate (Se^6+^)	50	5.825 ± 0.225	37.434 ± 3.394	138.938 ± 4.826
	350	4.938 ± 0.148	284.134 ± 5.811	163.685 ± 8.494
Selenourea	50	6.012 ± 0.101	101.244 ± 8.201	134.083 ± 3.653
	350	5.234 ± 0.178	485.622 ± 19.029	163.685 ± 8.494
Selenocysteine	50	4.781 ± 0.110	132.284 ± 10.753	145.052 ± 5.240
	350	1.828 ± 0.055	909.900 ± 22.261	173.054 ± 12.376
Selenomethionine	50	4.730 ± 0.186	188.064 ± 6.935	133.607 ± 5.112
	350	2.255 ± 0.121	626.167 ± 21.638	183.686 ± 7.454
Reference with 50 μM of K_2_SO_4_ (S^6+^)	0	6.116 ± 0.132	n.d. ^1^	115.678 ± 5.925

^1^ n.d.—not detectable

**Table 3:** Selenobiotin content and percentage of the total pool of biotin vitamers present in *Meyerozyma guilliermondii* ATCC 6260 cells cultured in selenium-supplemented 0S medium.

Selenium Form	Se Dose [μM/L]	Selenobiotin [μg/100 g DM]	Selenobiotin [% of Biotin Vitamers]
Selenite (Se^4+^)	50	1.078 ± 0.034	10.2
Selenate (Se^6+^)	50	1.564 ± 0.051	12.2
Selenourea	50	1.760 ± 0.069	16.5
Selenocysteine	50	1.856 ± 0.054	15.6
Selenomethionine	50	2.000 ± 0.046	17.3
Reference with 50 μM of K_2_SO_4_ (S^6+^)	0	n.d. ^1^	0

^1^ n.d.—not detectable.

**Table 4:** Selenobiotin content and percentage of the total pool of biotin vitamers present in *Trichosporon cutaneum* LOCK 0254 cells cultured in selenium-supplemented 0S medium.

Selenium form	Se dose [μM/L]	Selenobiotin [μg/100 g DM]	Selenobiotin [% of biotin vitamers]
Selenite (Se^4+^)	350	30.165 ± 0.974	22.1
Selenate (Se^6+^)	350	31.940 ± 0.239	21.3
Selenourea	350	34.911 ± 0.529	23.4
Selenocysteine	350	37.218 ± 1.088	25.6
Selenomethionine	350	52.543 ± 2.616	28.4
Reference with 50 μM of K_2_SO_4_ (S^6+^)	0	n.d. ^1^	0

^1^ n.d.—not detectable.

## Data Availability

A set of raw results is available on request from the authors.
